# Touch-induced face conditioning is mediated by genetic variation in opioid but not oxytocin receptors

**DOI:** 10.1038/s41598-018-27199-2

**Published:** 2018-06-13

**Authors:** Yu Fu, Emre Selcuk, Sarah R. Moore, Richard A. Depue

**Affiliations:** 1 Department of Human Development and Institute of Human Neuroscience, Cornell University, Ithaca, New York 14850 USA; 2 Present Address: Department of Psychology, Middle East Technical University, Ankara, 06800 Turkey

## Abstract

Soft touch possesses strong prosocial effects that facilitate social bonding and group cohesion in animals. Touch activates opioids (OP) and oxytocin (OXT), two neuromodulators involved in affiliative behaviors and social bonding. We examined whether touch serves as an unconditioned reward in affective conditioning of human faces, a basic process in social bonding, and whether this process is mediated by variation in mu-OP (*OPRM1*) and OXT (*rs53576*) receptor genes. Participants viewed affectively-neutral human faces, half of which were paired with a brief soft brushing on the forearm as an unconditioned stimulus (US). Paired and unpaired faces were rated for positive affective and sensory features of touch. Variation in *OPRM1* but not *rs53576* significantly modulated strength and development of conditioning, indicating that touch-induced mu-OP but not OXT activity provides rewarding properties of a US in conditioning. Implications for touch-induced *mu*-OP activity in normal and disordered conditioned social bonding are discussed.

## Introduction

The most effective and critical stimulus in the formation of mammalian pair and maternal-infant bonds is soft tactile stimulation^1,2^. Mammals have evolved a dedicated soft touch neural system that is separate from that mediating hard touch, and nuclei in this pathway are interconnected with reward circuitry and brain regions important for social bonding^3,4^. In humans, the amount of affectionate touch strongly correlates with satisfaction, reciprocity, and attachment experienced in intimate relationships^5^, and even brief touch from a friendly stranger can have marked prosocial effects including increased generosity^6^, compliance^7^, and altruism^8^, and decreased stress and aggression^9,10^.

One prosocial effect of touch is that it can be highly pleasurable. That soft touch induces a marked pleasurable affective state in humans has recently been demonstrated^3,4,11^. Furthermore, these pleasurable effects are sufficiently strong to serve as a reward in affective conditioning to neutral social contexts in both animals and possibly in humans^1,12–15^. In addition, touch can induce a state of pleasant physiological quiescence. In monkeys, allogrooming reduces heart rate and behavioral indices of stress. The animal who is being groomed can become so relaxed that it can quite literally fall asleep^1,16^. Similar calming, comforting effects of touch were also reported in human infants and adults^5,17–19^. Together, the pleasure and physiological quiescence induced by touch are hypothesized to provide the affective state that enhances (i) reward, as a basis of associative conditioning required for the formation and maintenance of selective social bonds, and (ii) relaxation, as a requisite condition of creating social trust and maintaining social bonds for a prolonged period^1,16,20,21^.

Another process in which soft touch likely plays a critical role in the formation and maintenance of social bonds is affective associative conditioning. When animals and humans experience naturally-rewarding stimuli, they learn to like and prefer previously neutral contexts and individuals present at the time of reward. Such conditioning is hypothesized to underlie the formation of selective social bonds, including pair and maternal-infant bonds^13,22^, and especially in humans, to serve as the basis of social memories that maintain those bonds over time and during periods of separation^20^.

Exactly which neuromodulators mediate the effects of soft touch on human social bonding is not yet established, but both endogenous opioids (OP) and oxytocin (OXT) play a significant role in animals. OP, mediated primarily by the mu-opioid receptor (MOR), facilitates consummatory reward, which is associated in humans with feelings of pleasure, gratification, and liking^20,23,24^. MOR activation facilitates vigorous grooming, play behavior, infant maternal clinging, maternal grooming of infants, and selective attachments in rodents (including prairie voles), sheep, and monkeys, as they are densely concentrated in brain regions associated with reward circuitry, and mediate the positive reinforcing properties of OP and the affective conditioning of maternal and contextual cues to reward^13,25–27^. Exogenously-injected MOR agonists and activation of MORs in brain reward regions serve as unconditioned rewarding stimuli that elicit self-administration, produce a conditioned place-preference, facilitate positive hedonic feelings similar to those activated by soft touch, and conditioned liking of contextual cues associated with reward^20,28^. These findings indicate that MORs mediate the rewarding effects that are necessary to establish the positive affective conditioning believed to underlie social bonding. Importantly, Case *et al*.^29^ provide the first direct support in humans for the hypothesis that opioids have a role in CT-mediated affective quality of touch.

OXT release and OXT receptor (OXTR) binding underlies several major processes that enhance social approach for affiliation. OXT, and to some extent OP, attenuates stress in response to social cues by markedly reducing amygdala arousal, autonomic nervous system reactivity, and stress hormones^30^. OXT thereby plays an important role as a biological mechanism for stress-protective effects of positive social interaction. OXT also enhances processes associated with social cognition and memory, including attention to and perception of socioemotional facial and bodily cues^30^. Most relevant to reward processing, OXT appears to orient reward circuits to social stimuli through interactions with dopamine (DA, a major neuromodulator that also facilitates reward processing) and OP systems. OXT neurons send projections to brain reward regions that contain DA and OP neurons and their projections, in which OXTR activation increases DA and OP release^31,32^. OXT does not, however, appear to mediate reward *by itself* because, although OXT receptors are found and may interact with mechanisms in the reward circuit, there is no literature that demonstrates that OXT *by itself* mediates effects of natural or drug reward, self-administration, or place conditioning in rats^33^. Moreover, in humans, intranasal OXT administration has shown no significant effects on positive or negative mood ratings^34–36^ or pleasantness of soft touch^37^.

Genetic variance in mu-OP and OXT receptors has been linked to individual differences in social behaviors that may reflect the differential roles of OP and OXT in facilitating human social bonding. The G allele of the single nucleotide polymorphism A118G within the human mu-opioid receptor gene (*OPRM1*) is associated with a gain-of-function. Both infant and mother rhesus macaques with the G allele in a single nucleotide polymorphism that is functionally similar to A118G in humans displayed more behaviors that maintain close physical proximity in mother-infant dyads^38,39^. In humans, G allele carriers had an increased tendency to become engaged in affectionate relationships and experienced more pleasure in parent-child interaction and in general social situations^40,41^, suggesting that the G allele may enhance the OP-mediated rewarding effects of social interaction. For OXT, mounting evidence indicates that a single nucleotide polymorphism located in the third intron of *OXTR*, *rs53576* (A/G), is associated with a G allele gain-of-function relevant to social bonding. In contrast, in humans the A allele is linked with increased amygdala activation to negative social stimuli^42^, lower responsiveness to the anxiolytic effect of social support^43^, lower levels of psychological resources^44^, and decreased parental sensitivity, empathy, expression of nonverbal affiliative cues, and prosociality^42,45–47^, all suggesting a reduction in OXT’s effects of stress-reduction, cognitive enhancement, and reward orientation during social interactions that support social bonding.

In the current study, based on recent integrative reviews^20,26^ and human studies with soft touch on the effects of OXT and OP^29,37^, we hypothesized that brief soft touch serves as an effective reward to induce positive affective conditioning towards neutral faces. Associative conditioning is used as a standard index of whether a stimulus (e.g., soft touch) serves as a reward in animal studies of conditioned place- and mate-preference^28^. In humans, it occurs in all sensory and cross-modal domains and with biologically significant stimuli (e.g., sweet food) in as few as *one* pairing of a neutral stimulus with reward^48^. Based on the potential differential effects of OP and OXT on social affective processes reviewed above, we also hypothesized that the prominent OP role in the pleasurable rewarding effects of soft touch that are necessary for affective conditioning in humans would be revealed by genetic variation in MOR. In contrast, we hypothesized that the facilitating role of OXT in social attention, cognition, and memory, but not specifically in reward, would render the effects of genetic variation in *OXTR* in affective conditioning insignificant. Specifically, we predicted that touch-induced conditioning is (i) enhanced in G allele (GG/AG) vs. A allele (AA) carriers of the *OPRM1* A118G polymorphism, but (ii) is not enhanced in G allele (GG), nor reduced in A allele (AA/AG), carriers of the *OXTR rs53576* polymorphism.

**Figure 1:**
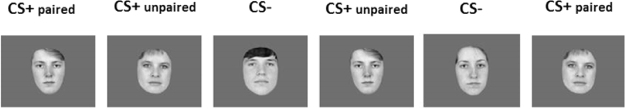
Illustration of the experimental design. Four emotionally-neutral faces (2 male, 2 female) used with permission in the study were selected from the series of faces developed by Matsumoto and Ekman^61^. [Available as the Japanese and Caucasian facial expressions of emotion and neutral faces (JACNeuF). D Matsumoto, P Ekman - Human Interaction Laboratory, University of California, 1988. (https://www.humintell.com/for-use-in-research/; ekmansf@itsa.ucsf.edu). For each participant, two faces (e.g., first and second in figure) were conditioned with forearm brush strokes during their presentation and therefore represent CS+ faces; the other two faces (e.g., the third and fifth in figure) were never paired with brushing and represent CS− faces. Faces assigned CS+ vs CS− status were randomized across participants, but each CS type consisted of a male and female face. The US-CS pairing accords to a 50% partial reinforcement: only half of the presentations of the CS+ ’s were accompanied by the US (e.g., first and last faces in figure [CS+ paired]) and half were not (e.g., second and fourth faces in figure [CS+ unpaired]. Affective ratings of CS+ faces were collected in unpaired trails and thus were uncontaminated by direct effects of the US.

To assess whether receptor genotype groups differed in the pleasantness of the US brushing, on a day after the conditioning study, participants twice rated the pleasantness of brushing using the same brushing method and the same affect rating scale as used in the conditioning portion of the study (Fig. S2). The two ratings were separated by a ten-minute period while saliva was collected. In the conditioning portion of the study, participants viewed four emotionally-neutral faces, and received brief gentle brushing (US) on their forearm as they viewed two of the faces (CS+); the other two faces were never brushed (CS−). Half of the CS+ trials were not paired with brushing (a 50% reinforcement schedule), and thus were uncontaminated by direct US effects. After each unpaired CS+ trial (and CS− trials), participants rated both the pleasantness (reflecting the rewarding affective feature of touch) and gentleness (reflecting the soft sensory feature of touch) of the face (see Figs 1, S1 and S2 for illustrations of the conditioning paradigm, brushing location, and affect rating scale, respectively). Modulation of conditioning was assessed as a function of genotype for the *OPRM1* A118G and OXT *rs53576* (A/G) single nucleotide polymorphisms.

## Results

For analyses, several subgroups for both *OPRM1* and *OXTR* were combined. For the *OPRM1* gene the G allele is the minor allele, while for *OXTR* the A allele is the minor allele. Both alleles are at low frequency in the population, meaning that unless in a very large sample, the size of the participant subgroups representing OP GG and OXT AA are typically very small. For instance, in this study, the OP GG subgroup had only 3 participants, while the OXT AA subgroup had only 12 participants; both are common frequency findings in the literature^49,50^. A study by Way *et al*.^51^ had a larger sample (n = 122) and so the OP GG genotype subgroup was large enough for reliable analyses of an additive genetic effect. However, when Way *et al*.^51^ assessed fMRI (in a smaller sample n = 33), the OP GG subgroup had only one individual, and so that subgroup was, as did we, combined with the OP AG genotype subgroup. Therefore, when sample size is relatively small, the homozygous and the heterozygous subgroups of the minor alleles are often combined in human and primate studies^38–41,46,52^. Thus, it is not statistically reliable or powerful enough to use groups of 3 and 12 to test additive model effects for OP and OXT in this study. Therefore, *OPRM1* GG and AG genotypes and *OXTR* AA and AG genotypes were combined for analyses.

The two participant ratings of the US brushing stimulus prior to the conditioning portion of the study were not found to differ for either the OP or OXT genotype groups [order × OP genotype: *F (1, 64)* = *1.338; p* = *0*.2*5*2; *partial η*^2^ = *0.02*. order × OXT genotype: *F (1, 64)* = *0.87*2*; p* = *0.354; partial η*^2^ = *0.013*] nor for the genotypes in interaction [order × OP × OXT: *F (1, 64)* = *0.058; p* = *0.81; partial η*^2^ = *0.001*] and so the two ratings were averaged. Moreover, neither between groups within receptor genotypes nor their interaction were significantly different [*OP*: *F (1, 64)* = *0.6*22*; p* = *0.433*; *partial η*^2^ = *0.01; OXT*: *F (1, 64)* = 2*.181; p* = *0.145*; *partial η*^2^ = *0.033; OP* × *OXT*: *F (1, 64)* = 2*.558; p* = *0.115; partial η*^2^ = *0.038*]. It could be that the rating measure is not sensitive enough to capture genotypic effects in US pleasantness, though the measure was sensitive enough to reveal genotypic differences in conditioning. It could also be that, while OP influences touch pleasantness^29^, the influence may not be manifested by differences *within* the *OPRM1* or *OXTR* genotypes.

Participant ratings of pleasant and gentle for each face on each rating trial, subtracting their respective baseline rating in the pre-conditioning familiarization presentations, served as the dependent variable. A mixed ANOVA using rating type (pleasant vs. gentle), CS type (CS+ vs. CS−), and trial order (1^st^ to 5^th^ brushing event) as within-subject variables, and using OP genotype (GG/AG vs. AA) and OXT genotype (AA/AG vs. GG) as between-subject variables, was performed. Different components of this overall ANOVA were used to answer a series of questions regarding conditioning efficacy and genotype effects.

### Efficacy of Affective Conditioning Procedure

**Figure 2:**
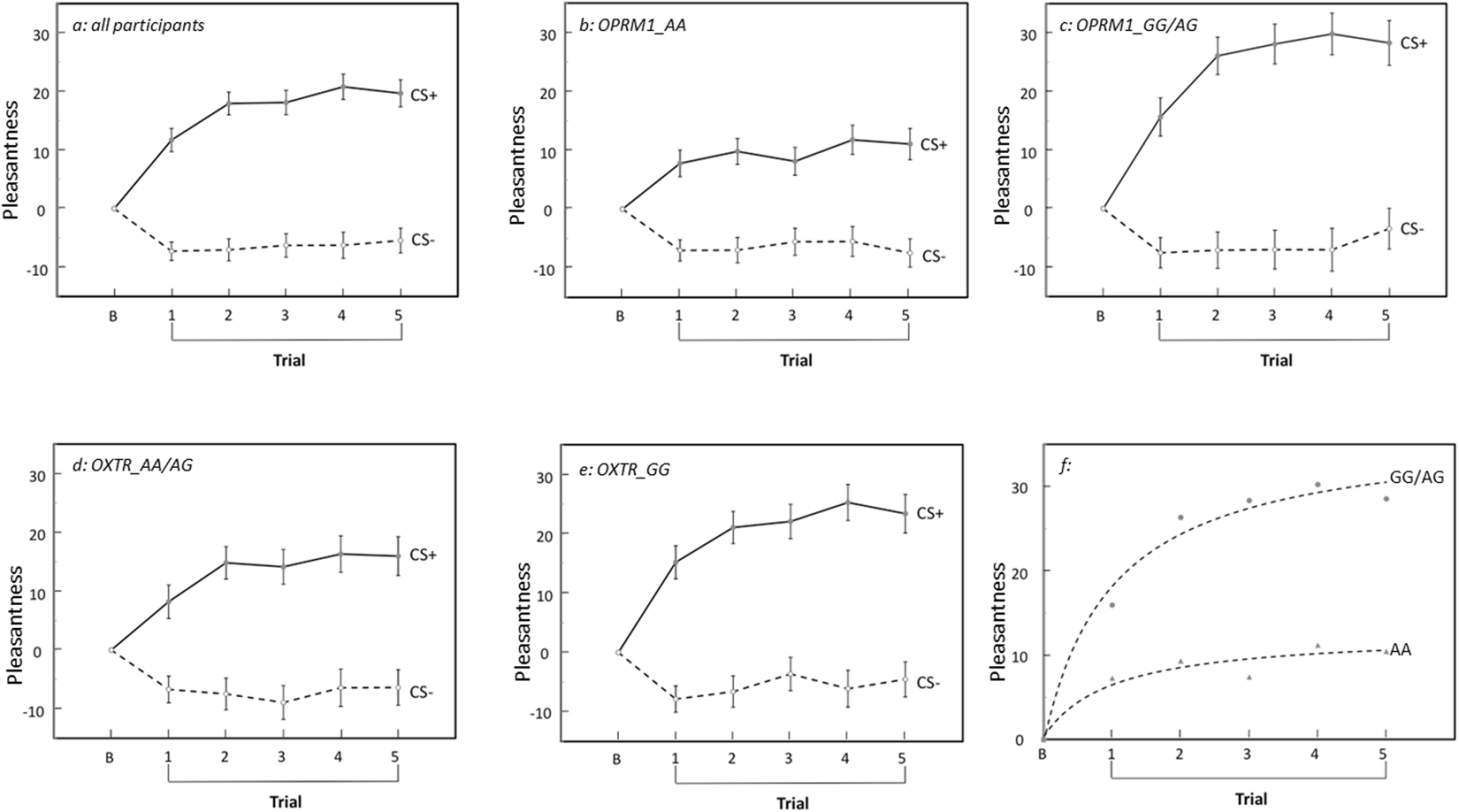
Affective conditioning across five trials as a function of *OPRM1* and *OXTR* genotype subgroups, and conditioning curves for the *OPRM1* genotype groups. *a* = all participants with all genotypes combined (N = 68); *b* = participants with AA *OPRM1* genotype (N = 46); *c* = participants with GG + AG *OPRM1* genotypes combined (N = 22); *d* = participants with AA + AG *OXTR* genotypes combined (N = 36); and *e* = participants with the GG *OXTR* genotype (N = 32); and *f* = curves fit to illustrate the pattern of the development of conditioning across trials in *OPRM1* AA vs. GG + AG genotype subgroups. Pleasantness on the y-axis represents an average of the pleasant and gentle ratings to faces, which did not statistically differ (see Results), minus the average of the baseline values for these ratings.

Brief touch-induced affective conditioning was indicated by a CS main effect [*F (1, 64)* = *109.687; p* < *0.001; partial η*^2^ = *0.63*2], where facial ratings of CS+ faces were significantly higher than for CS− faces. When facial ratings of CS+ and CS− faces were compared on each rating trail, after only one pairing with soft touch brushing, CS+ faces were already rated significantly higher than CS− faces, and this difference in ratings remained significant for all subsequent trials 2–5 [*F (1, 68) ranging from 57.885 to 87.085 for the 1*^*st*^ *to 5*^*th*^ *trial; all p values* < *0.001, with partial η*^2^ *values ranging from 0.460 to 0.56*2]. In addition, a significant CS*trial order two-way interaction [*F (4*, 2*56)* = *5.3*2*9; p* < *0.001; partial η*^2^ = *0.077*] indicated that the pattern of facial ratings also differed between CS+ and CS− faces across the five trials. Post-hoc analysis revealed that facial ratings for only the CS+ faces (but not the CS− faces) showed an increase across trials [*F (4*, 2*68)* = *7.196; p* < *0.001; partial η*^2^ = *0.097*], where the ratings of CS+ faces significantly differed from trial 1 ratings on trial 2 (*p* = *0.031*), 4 (*p* = *0.001*), and 5 (*p* = *0.005*) in pairwise comparisons. As illustrated in Fig. 2a and Table S1, this pattern indicates that the facial ratings of CS+ faces reflect an increasing development in the process of conditioning across time that is not evident in the pattern of CS− ratings.

Next, components of the ANOVA using rating type (pleasant vs. gentle), CS type (CS+ vs. CS−), and trial order (1^st^ to 5^th^ brushing event) as within-subject factors were used to determine if the pleasant and gentle facial ratings showed similar conditioning effects. The ANOVA indicated that the affective conditioning patterns were not significantly different for pleasant versus gentle ratings (Table S1): all levels of interaction terms containing rating type were non-significant (*all p values* > *0.20*). Thus, both the pleasant and gentle ratings similarly showed significant conditioning.

Finally, the efficacy of using two CS+ and two CS− faces, in addition to a 50% reinforcement schedule for CS+ faces, to eliminate contingency awareness of the US-CS conditioning procedure was assessed. Conditioning did not appear to be influenced by contingency awareness, as post-study interviews showed that no participant reported an awareness of an association between the occurrences of brushing and any specific faces, nor between brushing and liking of specific faces, results that replicate those of many studies of affective conditioning^48^ and of those found by Buchel *et al*.^53^ using a conditioning procedure similar to the current one.

### Effects of opioid and oxytocin genotypes on affective conditioning

The entire sample of participants was used as one group since the sample is homogeneous in terms of age, sex, and socioeconomic (SES) background, and therefore no consistent effect on genotype frequencies across sample subgroups is expected. Some studies^54^ have found a higher frequency of the *OPRM1* G allele in Asian compared to Caucasian populations. However, because of the small size of the Asian subgroup in the current sample (N = 11 of 68, 16%), an overall ANOVA model with ethnic group added as an additional variable to the current model did not have enough participants in all combinations of design cells, and thus could not be reliably estimated. In order to evaluate possible effects of ethnic group on affective conditioning, the same analyses performed above were run on only the Caucasian subgroup in the sample (57 of 68, 84%). For both the full sample and the Caucasian subgroup, no main effect or interaction of the *OXTR* genotype was significant in the ANOVA (*all p values* > *0.05*), indicating that the *OXTR* genotype did not modify conditioning results to any large extent (see Results and Fig. 2e,f). In contrast, as shown in Fig. 2b,c and Table S1, a significant main effect of *OPRM1* genotype [*full sample****:**** F(1, 64)* = *8*.2*46; p* = *0.006, partial η*^2^ = *0.114;Caucasian subgroup: F(1, 53)* = *6.83*2*; p* = *0.01*2*; partial η*^2^ = *0.114*] showed that participants with GG/AG genotypes of the *OPRM1* gene provided higher facial ratings than those with the AA genotype. Thus, in general, the results for the Caucasian subgroup were statistically not different from those derived from all participants, suggesting that, in this study, ethnic group did not influence affective conditioning.

Analyses on the total group of participants showed that OP but not OXT genotypes modulated conditioning. As shown in Fig. 2b,c and Table S1, a significant main effect of OP genotype [*F(1, 64)* = *8*.2*46; p* = *0.006, partial η*^*2*^ = *0.114*] showed that participants with GG/AG genotypes of the *OPRM1* gene provided higher facial ratings than those with the AA genotype. Further, a two-way interaction between *OPRM1* genotype and CS type [*F (1, 64)* = *11.787; p* = *0.001; partial η*^*2*^ = *0.156*] showed that this rating difference for GG/AG vs. AA genotypes occurred only for CS+ faces [*F (1, 66)* = *19.579, p* < *0.001, partial η*^*2*^ = *0.229*], but not for CS− faces [*F (1, 66)* = *0.007, p* = *0.933, partial η*^*2*^ < *0.001*]. In contrast, no main effect for *OXTR* genotype on conditioning (*F (1, 66)* = *2.581, p* = *0.113, partial η*^*2*^ = *0.039*) or interaction of the *OXTR* genotype with type of rating (*F (1, 66)* = *1.908, p* = *0.172, partial η*^*2*^ = *0.029*), type of CS (*F (1, 66)* = *1.738, p* = *0.192, partial η*^*2*^ = *0.026*), or *OPMR1* genotype (*F (1, 66)* = *0.043, p* = *0.836, partial η*^*2*^ = *0.001*) were significant, indicating that *OXTR* genotype did not modify conditioning results to any large extent (see Fig. 2d,e). Thus, OP genotype specifically influenced ratings for faces that were paired with soft touch reward, and accounted for ~23% of the variance in conditioning.

OP genotype also modulated the development of conditioning, as illustrated by a three-way interaction between OP genotype, CS type, and trial order [*F (4, 256)* = *3.899; p* = *0.004; partial η*^*2*^ = *0.057]*. As illustrated in Fig. 2f, for the OP AA genotype, there was only a CS main effect for facial ratings [*F (1, 45)* = *61.278; p* < *0.001; partial η*^*2*^ = *0.577*], but no trial order main effect [*F (3.018, 138.638)* = *1.247; p* = *0.293; partial η*^*2*^ = *0.027*] nor CS*trial order interaction [*F (4, 180)* = *2.339; p* = *0.057; partial η*^*2*^ = *0.049*], thus showing no development of conditioning across trials. In contrast, as shown also in Fig. 2f, in the OP GG/AG genotype group, in addition to a significant CS main effect [*F (1, 21)* = *40.181; p* < *0.001; partial η*^*2*^ = *0.657*], there was a significant trial order main effect [*F (2.009, 42.181)* = *4.755; p* = *0.002; partial η*^*2*^ = *0.185*] and a significant CS*trial order interaction [*F (4, 84)* = *4.141; p* = *0.004; partial η*^*2*^ = *0.165*].

Further analysis of the OP GG/AG data showed that a simple main effect of trial order was evident only for CS+ [*F (2.543, 53.412)* = *7.585, p* = *0.001, partial η*^*2*^ = *0.265*] but not for CS− faces [*F (2.534, 53.209)* = *0.783, p* = *0.489, partial η*^*2*^ = *0.036*]. Pairwise comparisons confirmed that facial ratings of CS+ faces on trial 1 were significantly lower than on trials 2–5 (*all p values* < *0.05*). Thus, ratings continued to increase across trials 1–5, but they were not significantly different after trial 2, as indicated by no difference in facial ratings between combinations of trials 2–5 (*all p values* > *0.05*).

As shown in Fig. 2f, these different patterns of development of conditioning as a function of OP genotype were further quantified by fitting curves to the means of facial ratings for CS+ faces across each of all five trials, with the baseline point included. Only the mean of the ratings was used for curve fitting, as only a description of the different patterns of development of conditioning in OP groups was intended, rather than an estimation of the variance of the curve fit. For both OP genotype groups, an inverse curve fit the means of the CS+ trial ratings significantly (*p* < *0.001* for GG/AG group; *p* = *0.003* for AA group), confirming a decelerating increase of ratings across trials, a pattern that is a hallmark of the process of conditioning over time^55,56^. However, as shown in Fig. 2f, in the OP AA genotype group, the curve leveled off quickly after the first brushing trial, while in the OP GG/AG genotype group, an asymptote was not approached until after trial 2. These findings together indicate a much stronger and more enduring process of conditioning over trials in the OP GG/AG group than in the OP AA group.

The genotype effects on affective conditioning were not significantly different for pleasant versus gentle ratings (Table S1): interaction of all levels that contains rating type, CS type, trial order, OP genotype, and OXT genotype were not significant (*all p values* > *0.05*).

## Discussion

We observed that soft touch induced positive affective conditioning of affectively-neutral human faces. The conditioning effects were multifaceted, enhancing facial ratings reflecting both the affective and sensory aspects of the US. Individual differences in the *OPRM1* gene (A118G) significantly modulated conditioning, where G allele carriers (GG/AG) showed enhanced conditioning relative to individuals with the AA genotype. In contrast, variation in the *OXTR* gene (*rs53576*) had no significant influence on conditioning in this study. The current results suggest that brief soft touch can promote the formation of human liking of social stimuli through a multifaceted affective conditioning process, and that mu-OP activity plays an essential role in mediating this conditioning.

The affective conditioning procedures produced robust conditioning that often began after only one pairing of a face with soft touch, indicated by significantly increased pleasantness and gentle ratings of faces paired with touch (CS+) compared to unpaired faces (CS−). Moreover, conditioning of CS+ faces showed a clear increase across brushing trials, demonstrating a robust development of conditioning across CS+ -US pairings. Hence, the results indicate that soft brushing appears to have a strong rewarding effect that is capable of mediating affective conditioning to a human face.

These findings are consistent with many animal studies and with previous human studies showing that touch can induce conditioned attention and liking to a neutral odor^12,14^. The current study extends touch-induced affective conditioning to social stimuli. Further, in contrast to the US adopted in previous animal and human studies that mimic the touch experienced in socio-sexual relationships, such as maternal licking and grooming in rats, grooming in nonhuman primates, and massage in humans, the US in the current study was a brief soft touch that can be experienced both in close relationships and in friendly social contact. This soft brushing activates a type of C-tactile fiber that is widely distributed in human hairy skin and may provide a peripheral mechanism for conveying the positive hedonic feeling of tactile contact with conspecifics, thereby promoting affiliative behaviors^3^. Thus, the conditioning effect demonstrated here may provide an underlying mechanism through which touch promotes bonding and group cohesion in all types of human relationships. In support of this idea, the rapid acquisition of positive social perception towards CS+ faces found here is concordant with observations that individuals who receive very brief touch behave more generously, compliantly, and altruistically towards an unfamiliar person who provided the touch^6–8^. Taken together, the human conditioning paradigm used here may be useful in further studies of the effects of soft touch in social behavior and bonding.

OP genotype significantly modulated touch-induced conditioning. Participants with GG/AG genotypes of the *OPRM1* gene conditioned more rapidly and provided higher facial ratings than those with the AA genotype, and this effect occurred only for CS+ faces, but not for CS− faces. Thus, OP genotype specifically influenced ratings for faces that were paired with soft touch reward. Furthermore, OP genotype also modulated the development of conditioning. Whereas the OP AA genotype showed no development of conditioning across trials, the OP GG/AG genotypes demonstrated a highly significant development of conditioning of facial ratings across brushing trials 1–5. Moreover, for both OP genotype groups, an inverse curve fit the means of the CS+ trial ratings significantly, confirming a decelerating increase of ratings across trials, a pattern that is a hallmark of the process of conditioning over time^55,56^. However, in the OP AA genotype group, the curve leveled off quickly after the first brushing trial, while in the OP GG/AG genotype group, an asymptote was not approached until trial 3 or later. These findings indicate a much stronger and more enduring process of conditioning over trials in the OP GG/AG group.

The significant effects of OP genotype on touch-induced affective conditioning are consistent with (i) an OP mediation of the rewarding property of soft touch in animals and humans^20,26,29^, and (ii) that the 118G allele has a gain-of-function of MOR activity and is associated with an increased tendency to engage in social affiliation and to experience social reward^38,40,41^. In contrast, genetic variance in the *OXTR* gene (*rs53576*) did not have a significant effect on magnitude or development of conditioning. This is consistent with some previous findings that OXT orients reward systems towards social stimuli without directly mediating reward *per se*^33–36^. The facilitatory role of OXT may be more predominant in the approach to or initiation of social reward in humans (vs. rodents) than in the consumption of it^1,22^. For instance, OXT release is increased and facilitates the initiation of maternal behaviors and formation of pair bonding, but it may not be effective in the maintenance of these affiliative behaviors in humans^13,22^. OXT also interacts differently with DA and OP systems. With DA, an important neuromodulator for *appetitive-incentive* reward, OXT projections to DA neurons in the ventral tegmental area enhance DA release, and the activation of DA neurons also increases OXT release, suggesting a positive-feedback loop between the two that mutually facilitates approach for affiliation^32^. On the other hand, in the case of OP, the main neuromodulator for *consummatory* reward^13,24^, OXT projections to OP neurons in the arcuate nucleus also increase OP release, but some studies indicate that OP activation inhibits OXT activity, suggesting that the effects of OP in bringing affiliative interactions to a gratifying conclusion may involve suppressing OXT-facilitated initiation of affiliative behaviors^20^. As administration of soft touch in the current study activates a consummatory phase of reward, the current result that OXT had no significant effect on soft touch-induced conditioning is not only consistent with the lack of OXT on pleasantness ratings of soft touch^37^ and also may help to clarify differential roles of OXT and OP in social bonding.

One finding of this study may contribute to a fuller understanding of the conditioned formation and maintenance of social bonds. Pleasant and gentle ratings had similar conditioning patterns, suggesting that not only the affective (pleasantness) but also the sensory (gentle, softness) aspects of soft touch were conditioned to previously neutral faces^57^. This raises the possibility that the two steps involved in touch-induced conditioning to social stimuli – the association of feelings of the US to the CS, and the formation of a social perception based on that conditioned feeling – are both embodied, and thereby possess the multifaceted feature of sensory information in addition to affect. This multifaceted affective association is consistent with embodied cognition research, which shows that social judgment is largely embodied and can be significantly influenced by the momentary sensorimotor experience, such as tactile stimulation^58^, and it may well contribute to the diversity and complexity of social perceptions that are formed in relationships. Moreover, the similar pattern found here of OP genotype mediation for both pleasant and gentle ratings of the conditioned stimulus suggests that the OP-enhanced reward encoding of soft touch may include multiple aspects of the US. As sensory processing of emotional stimuli is enhanced through increased attention and brain activation in sensory cortex^59^, besides mediating the rewarding value of soft touch, MOR activation may also facilitate its sensory processing, deepening the multifaceted encoding of the US and thus its conditioning to the CS. As G allele carriers may have enhanced MOR activity, these effects may be stronger for them.

As with any gene association study, the current results should be considered suggestive until replicated. However, several aspects of the study increase the reliability of its findings. First, the selection of OP and OXT candidate genes was based on their strong empirically-based relation to affiliative behavior, a basis that has also generated a theoretical framework for the effect of these genes in social bonding. Thus, the genes selected for study were theoretically- and empirically-determined on the basis of a robust formulation of their relation to social bonding processes. Second, in contrast to most candidate gene investigations which assess only correlations between genes and *un-manipulated* variables, our study adds to a line of *experimental* studies that illustrate the gain in power that comes from measuring theoretically-relevant quantitative processes under experimental conditions and from applying statistical models to test specific *a priori* hypotheses^46,50^. Indeed, in this study, OP genotype accounted for 23% of the variance in experimentally-measured affective conditioning (but see below), an effect size that well exceeds the range (5–10% of variance) expected for complex, polygenic traits^38^. Finally, the current study assessed two genes (*OPRM1* and *OXTR rs53576*) that are relevant to soft touch and affective conditioning processes, and demonstrated a substantial difference in effect between the two that is consistent with our hypotheses based on empirical and theoretical grounds.

Despite these supportive aspects of our candidate gene results, it is likely that the high variance accounted for by OP genotype in this study will be less in future studies. How much less is not possible to predict. It is still controversial whether the effect size from candidate gene studies are comparable, especially when these studies are based on *a prior* hypotheses stemming from the biological function underlying the studied phenotype. In these cases, one would expect the effect size to be larger, because the studied phenotype is more closely related to the function of the candidate gene. This may be a reason that, in studies focusing on A118G in humans and C77G in primates, effect sizes are larger than 1%, and as the studied phenotype became more specific, from psychometric scaling to specific OP function related to a specific behavior, the effect size increased from 3% to 5–10%^38,41^. Moreover, in this study, variance that is not relevant to the function of the candidate gene was controlled by carefully selecting and designing the studied phenotype. Thus, in the current study, the large effect size may be partially attributed to the fact that the touch-induced conditioning to CS+ faces is closely related to OP functioning that depends on the *OPRM1* genotype. In future studies, the magnitude of these effects, however, will depend on the extent to which the applied brushing methods activate the OP reward system, and to which this reward induces a robust conditioning effect. In a replication where methods vary, for instance where brushing is less pleasant or the conditioning effect is less prominent, we may expect the effect size of the *OPRM1* genotype to be reduced. In addition, as effect size often drops with larger sample sizes, a replication with larger sample sizes may be expected to produce smaller effect sizes.

To our knowledge, this is the first human study that includes both OP and OXT genotype variations in processes related to human bonding. However, it is important to emphasize that the current study does not rule out a role for OXT in social conditioning, but rather that its magnitude of effect in mediating touch-induced reward in affective conditioning appears to be small, accounting for only a small proportion of variance in conditioning. It is possible that the sample size of this study provided insufficient power to detect a significant effect of *OXTR* variation on conditioning. Moreover, the paradigm used in the study focused on the role of touch-induced reward in conditioning, but not other social processes that are perhaps affected more by OXT activity. For instance, as the current affective conditioning paradigm does not introduce components of stress, social cognition or social approach – all of which relate to OXT functioning in social bonding – the conditioning effect may be largely determined by receiving the consummatory reward of soft touch, which is mediated by OP functioning. This may explain the observed large effect of OP genotype. Therefore, this single dissociation in our view is not a de-emphasis of the significance of OXT functioning in social bonding; instead, it implies the importance of differentiating the roles of various neurotransmitters in the dynamic and complex process of social bonding. Future studies involving double dissociation, e.g., investigating the effects of OP and OXT in two paradigms that emphasize the presumed different functions of OP or OXT would further explicate the differential roles of OP and OXT in social bonding.

In conclusion, the current study demonstrated soft touch-induced multifaceted affective conditioning to neutral faces, which was modulated by OP but not OXT genotypes. The findings raise the possibility that the subtle experience of brief soft touch in everyday social interaction may promote social bonding and cohesion through conditioned changes of social perception. Results also imply that alteration of OP functioning, such as those induced by genetic variance, early experience, and administration of OP-related drugs, could influence the experience of consummatory reward in social interaction and thus modulate the capacity for social affiliation in both nonclinical and socially-disordered populations.

## Methods

All methods in the study were carried out in accordance with relevant guidelines and regulations.

All protocols in this study were approved by the Cornell University Institutional Review

Board for Human Participants.

Written informed consent was obtained from all participants.

### Participants

Participants were 68 university females (19–23 years of age, all of middle to high socioeconomic status) selected at random from a larger pool (N = 450) of potential participants. Females serve as the model system for soft touch, since they likely evolved greater sensitivity for it and in general scored significantly higher in trait social closeness^60^. By using females, we maximize the effects of soft touch on affective conditioning as the initial demonstration that soft touch induces affective conditioning. Of the 68 participants, 57 (84%) were European Caucasian, 11 (16%) were East Asian (see Results for comparison of these two subgroups in conditioning). Participants were free of medical and psychiatric disorders.

### Genotyping

DNA was obtained from 0.5 ml of saliva using the prep-IT L2P protocol (DNA Genotek, Ottawa) and quantified using Qubit assay kits (Life Technologies). Allelic discrimination assays for *OPRM1* (rs1799971) and *OXTR* (rs53576) were performed by 5′ nuclease assays (Taqman SNP Genotyping Assays, Applied Biosystems Inc.) according to manufacturer recommendations using the Taqman Genotyping master mix (Applied Biosystems Inc.). The allelic discrimination assays were analyzed by real-time PCR using a Viia7 instrument and Viia7 software (Applied Biosystems Inc.). All DNA samples were genotyped for each locus in at least three replicate genotyping assays in the Genomics Facility, Institute of Biotechnology, Cornell University, Ithaca, New York.

Genotype frequencies for *OPRM1* were GG = 3 (4%), GA = 19 (28%), AA = 46 (68%), and for *OXTR rs53576* were GG = 32 (47%), GA = 24 (35%), AA = 12 (18%). Neither of the genotype distributions of *OPRM1 rs1799971* and *OXTR rs53576* deviated from Hardy–Weinberg equilibrium (P’s > 0.05).

### Stimuli

Four faces of young adults (2 male, 2 female) were selected from the neutral face database developed by Matsumoto and Ekman^61^. Outer features of the faces such as the ears and parts of the hair were removed, and the faces were presented in grayscale (Fig. 1). The four faces were presented and rated affectively in random order in a pre-conditioning familiarization phase in this study to habituate participants to the novelty of the faces and to obtain their baseline ratings before conditioning.

### Brushing

Similar to previous human studies^62^, participants sat with their non-dominant arm behind a screen and facing a computer monitor that presented face stimuli. An experimenter sat silently on the other side of the screen, out of sight of the participant, facing their own computer monitor, which signaled experimenter to brush during a trial by displaying a picture of a brush. Thus the experimenter was blind of the pairing of brushing and faces. When signaled, the experimenter applied two serial brush strokes with a soft, goat’s hair brush (*Da Vinci* Series 550 Black Goat’s Hair Wash Mottler brush, measuring 73 mm wide and 32 mm long), moving proximal (elbow) to distal (wrist) on the hairy skin of the medial surface of the forearm where C-tactile afferents were located^62^ (Fig. S2). Two repeated brushings have been found not to lead to affective insensitivity to the brushing^63^, and this width of brush at a velocity of ~3 cm/s was found to be highly effective in stimulating C-tactile fibers of the forearm and in eliciting high pleasantness ratings^16,63,64^ and GSR responses^53^.

In a constant room temperature of 23 degrees C, brush strokes were applied at the rate of ~3 cm/s with a normal force of ~0.4N, both of which are optimal for stimulating soft touch C-tactile fibers^4,62,65^. A relatively prolonged skin contact of the brush is also desirable for stimulating C-tactile fibers. Hence, the beginning (elbow) and end (wrist) points of a 12 cm distance were marked with a pen on the participant’s non-dominant forearm, which served as a guide for application of the brush strokes. Thus, each brush stroke was 4 s (~3 cm/s) in duration, ending with a gradual up-sweep of the brush. Experimenters were trained for a prolonged period in order to master the correct velocity and pressure (assessed on an electronic, ultrasensitive pressure gauge) of the brush stroke, and were calibrated on these variables twice weekly during the study.

### Conditioning

Affective conditioning consisted of pairing the forearm soft brush strokes (US) with emotionally neutral human faces (CS). In order to prevent conscious awareness of the conditioning process, two procedures were used: (i) two faces were paired with the US (CS+’s) and two were not (CS−’s); and (ii) 50% partial reinforcement was used, only half of the presentations of the CS+’s were accompanied by the US (CS+ paired) and half were not (CS+ unpaired). Affective ratings for CS+ faces were collected only from CS+ unpaired trials. Thus, pairing 50% of CS+ presentations with brushing also has the advantage of enabling analysis of affective responses to CS+ faces on trials *not* paired with the US (CS+ unpaired), and thereby uncontaminated by direct effects of the US^53,66^. Faces assigned CS+ vs CS− status were randomized across participants, but each CS type consisted of a male and female face. Fig. 1 shows an illustration of presentation paradigm.

There were 40 conditioning trials: 5 trials for each CS+ paired and CS+ unpaired male and female faces (i.e., 20 trials), where only the CS+ unpaired faces were rated affectively; and 10 trials for each of the CS− male and female faces (i.e., 20 trials), where only half (randomly selected) of the CS− trials were rated affectively. The order of trials was randomized with some adjustments ensuring that (i) each CS+ face was rated before its next pairing with the US so that the development of conditioning after each pairing can be assessed, and (ii) each brushing was separated by at least two trials, which prevents fatigue of C-tactile fibers^62,65^.

Each trial had the following sequence: (i) a computer *beep* indicated the initiation of a trial, (ii) 2 s delay, (iii) a face presentation for 9 s, (iv) brushing on CS+ paired trials (brushing began after the face appeared and continued until the face disappeared), and on rating trials (v) following face presentation affective ratings on scales shown on computer monitor, after which the next trial began with a *beep*.

### Affect rating scale

Facial ratings of (i) pleasant and (ii) gentle were performed on computer monitor using a visual analog scale ranging from 0 to 100, which was found to be highly effective in human studies of affective responses to brushing^62^. Both ratings were included in order to access the social perception of a face that is based on the bodily feelings evoked by viewing it, which are transferred from the bodily feelings generated by brushing. The pleasant rating assesses the valence of the face, reflecting the affective component of brushing. The gentle rating may assess the quality of softness and tenderness of the face. As an exploration to see if the sensory property of touch can be conditioned to a face, we selected one sensory aspect of the US: *softness*. We considered “gentle” as a word that reflects this aspect but is more relevant for describing human face characteristics. Indeed, the gentle rating may assess the quality of softness and tenderness of the face: we found in earlier exploratory studies that these three adjectives were equally highly represented in participant reports on the nature of soft brushing and of faces. Thus, softness possibly reflects a sensory aspect of brushing, as our social concept of gentleness may be scaffolded from early experience of touch softness.

Because inter-individual variation in interpretation and use of visual analog scales is reduced by use of (i) regular demarcations along the range of the scale, and (ii) the use of adjectival markers associated with the demarcations^67^, these were incorporated in the scale used in this study based on facial rating pilot data collected from a separate group of participants (Fig. S2 shows the Pleasantness scale).

### Analyses

Participant ratings of pleasant and gentle for each face on each rating trial, subtracting their respective baseline rating in the pre-conditioning familiarization presentations, served as the dependent variable. Using SPSS repeated measures, several mixed ANOVAs using rating type (pleasant vs. gentle), CS type (CS+ vs. CS−), and trial order (1^st^ to 5^th^ brushing event) as within-subject variables, and using OP genotype (GG/AG vs. AA) and OXT genotype (AA/AG vs. GG) as between-subject variables, were performed. Different components of these overall ANOVAs were used to answer a series of questions regarding conditioning efficacy and genotype effects.

## Electronic supplementary material

Supplementary Information
