# Supplementary material for: Touch-induced face conditioning is mediated by genetic variation in opioid but not oxytocin receptors
Source: Sci Rep. 2018 Jun 13;8:9004. doi: 10.1038/s41598-018-27199-2 (PMC5998070; doi:10.1038/s41598-018-27199-2)
Supplement: Supplementary file 1 — Supplementary Information [file 41598_2018_27199_MOESM1_ESM.docx]

***Supplemental Information***

**Touch-induced face conditioning is mediated by genetic variation in opioid but not oxytocin receptors SREP-17-52927A**

Yu Fu, Emre Selcuk, Sarah R. Moore, Richard A. Depue

*Department of Human Development and Institute of Human Neuroscience*

*Cornell University, Ithaca, New York 14850*

Table S1. Mean and Standard deviations of facial ratings

|  | Pleasant *M(SD)* | | | | | Gentle *M(SD)* | | | | |
| --- | --- | --- | --- | --- | --- | --- | --- | --- | --- | --- |
|  | Trial 1 | Trial 2 | Trial 3 | Trial 4 | Trial 5 | Trial 1 | Trial 2 | Trial 3 | Trial 4 | Trial 5 |
|  |  |  |  |  |  |  |  |  |  |  |
| All Participants  (N = 68) |  |  |  |  |  |  |  |  |  |  |
| CS+ | 9.70 (18.55) | 15.44 (19.44) | 14.92 (22.16) | 17.94 (22.09) | 17.40 (21.42) | 10.44 (17.31) | 14.19 (18.15) | 13.44 (19.69) | 16.75 (20.14) | 15.17 (21.80) |
| CS- | -6.41 (12.69) | -5.48 (15.76) | -3.89 (17.42) | -4.00 (17.96) | -3.72 (17.42) | -8.15 (16.01) | -8.85 (16.51) | -8.45 (16.32) | -8.44 (19.58) | -9.13 (17.85) |
| *OPRM1* AA  (N = 46) |  |  |  |  |  |  |  |  |  |  |
| CS+ | 5.94 (17.00) | 8.25 (15.11) | 6.44 (18.34) | 10.67 (18.93) | 10.38 (17.27) | 8.59 (16.21) | 10.38 (15.54) | 8.39 (16.59) | 11.69 (17.28) | 10.48 (18.02) |
| CS- | -6.53 (13.03) | -5.66 (15.51) | -3.49 (17.82) | -3.49 (17.82) | -5.14 (16.78) | -7.63 (14.52) | -8.69 (16.50) | -8.30 (15.42) | -7.67 (17.73) | -10.50 (15.87) |
| *OPRM1* GG/AG  (N = 22 ) | |  |  |  |  |  |  |  |  |  |
| CS+ | 17.54 (19.59) | 30.47 (19.15) | 32.66 (18.92) | 33.14 (20.77) | 32.09 (22.16) | 14.31 (19.23) | 22.17 (20.84) | 24.00 (21.78) | 27.32 (21.93) | 24.97 (25.90) |
| CS- | -6.16 (12.24) | -5.10 (16.64) | -4.74 (16.94) | -4.74 (16.94) | -0.77 (18.75) | -9.23 (19.09) | -9.20 (16.92) | -8.77 (18.44) | -10.05 (23.35) | -6.26 (21.53) |
| *OXTR* AA/AG  (N = 36) |  |  |  |  |  |  |  |  |  |  |
| CS+ | 4.88 (17.49) | 10.67 (18.30) | 7.93 (21.28) | 11.26 (20.34) | 11.34 (19.81) | 7.74 (17.57) | 11.24 (18.59) | 10.22 (19.81) | 13.35 (18.10) | 11.94 (18.66) |
| CS- | -6.49 (13.96) | -6.09 (15.29) | -7.36 (13.66) | -5.24 (16.55) | -5.56 (14.64) | -7.65 (13.61) | -9.73 (15.17) | -9.81 (12.16) | -8.57 (18.67) | -10.62 (17.92) |
| *OXTR* GG  (N = 32 ) |  |  |  |  |  |  |  |  |  |  |
| CS+ | 15.12 (18.48) | 20.80 (19.57) | 22.79 (20.72) | 25.45 (21.86) | 24.23 (21.40) | 13.48 (16.76) | 17.52 (17.31) | 17.06 (19.22) | 20.56 (21.88) | 18.79 (24.66) |
| CS- | -6.32 (11.32) | -4.79 (16.50) | 0.01 (20.39) | -2.61 (19.61) | -1.66 (20.15) | -8.71 (18.56) | -7.87 (18.10) | -6.93 (20.10) | -8.29 (20.85) | -7.46 (17.90) |
|  |  |  |  |  |  |  |  |  |  |  |


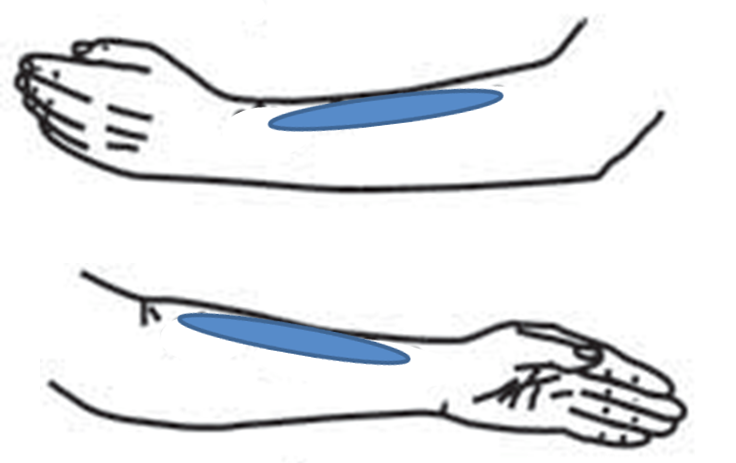


Figure S1. Illustration of the brushing location on forearm (adapted from ^50^). Two brush strokes were applied moving proximal (elbow) to distal (wrist) for a distance of 12 cm on the hairy skin of the medial surface of the forearm where C-tactile afferents were located (shaded ovals). The first stroke was applied on the outer edge of the forearm, and the second stroke was applied on the inner edge.


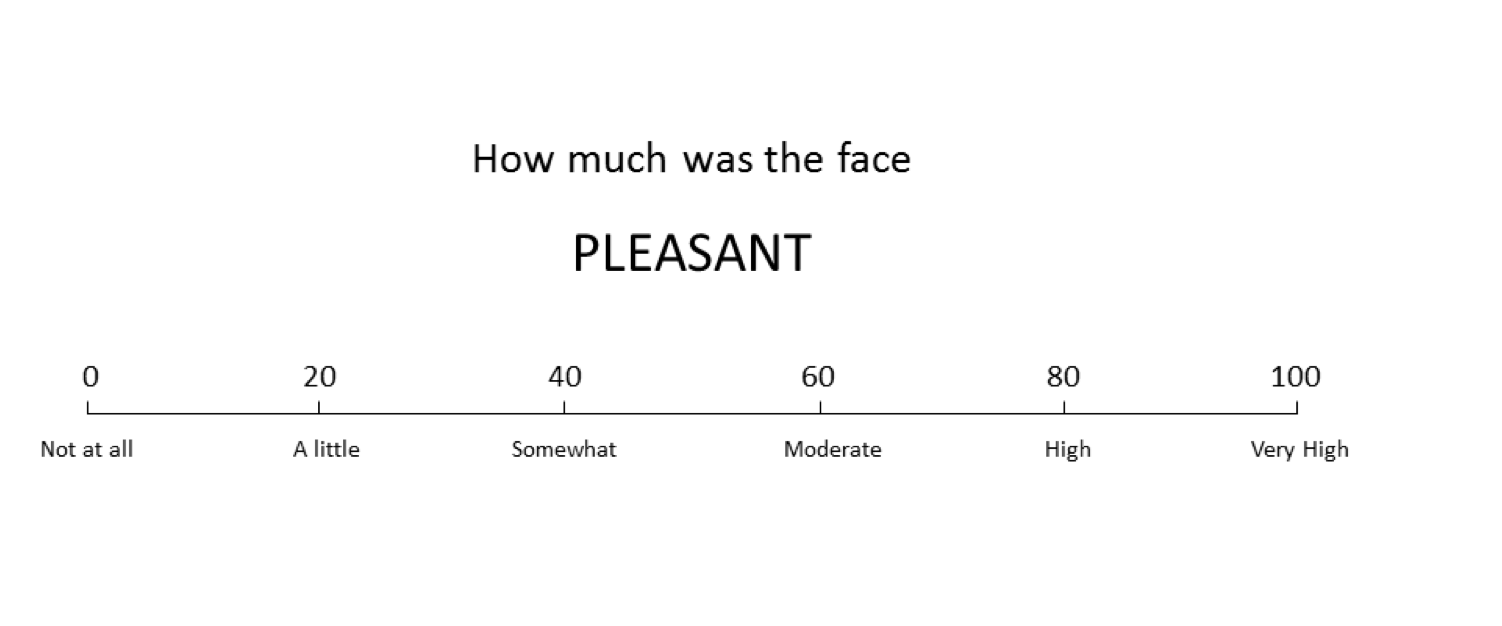
Figure S2. Illustration of the pleasantness rating scale. Ratings were performed on computer monitor using a visual analog scale ranging from 0 to 100. Twenty-point demarcations were grounded with adjectival markers indicating intensity of affective experience. Gentle ratings were performed following the pleasantness rating.
